# TCM Pattern Questionnaire for Lateral Elbow Pain: Development of an Instrument via a Delphi Process

**DOI:** 10.1155/2016/7034759

**Published:** 2016-07-20

**Authors:** Marcus Gadau, Shi-Ping Zhang, Wing-Fai Yeung, Zhao-Xiang Bian, Ai-Ping Lu

**Affiliations:** ^1^School of Chinese Medicine, Hong Kong Baptist University, 7 Baptist University Road, Kowloon Tong, Hong Kong; ^2^School of Nursing, The Hong Kong Polytechnic University, Hung Hom, Hong Kong

## Abstract

Individualized acupuncture treatment has been practiced for pain therapy. This study used acupuncture treatment for lateral elbow pain (LEP) as an example to study the diagnostic practice of individualized acupuncture treatment. A provisional version of LEP pattern questionnaire was developed based on a recent systematic review on TCM pattern diagnosis for LEP. A Delphi panel of 33 clinical experts from seven different countries was formed, and the Delphi survey was conducted in Chinese and English language for two rounds. Consensus was achieved from all 26 panelists who responded to the second round on 243 items of the instrument, which included a 72-question-long questionnaire. The mean level of expert consensus on the items of the final questionnaire was 85%. Consensus was found on four TCM patterns that could underlie LEP, namely, the* wind-cold-dampness pattern*, the* qi stagnation and blood stasis pattern*, the* dual deficiency of qi and blood pattern,* and the* retained dampness-heat pattern.* A list of signs and symptoms indicating one of the four TCM patterns and a list of preferred treatment modalities for each pattern were also generated. Our instrument shows considerable content validity. Further validity and reliability studies are under way.

## 1. Background

Personalized medicine has become the new trend in modern medical care [1–3]. Traditional Chinese medicine (TCM) has developed and used a sophisticated system of individualized medicine in the form of pattern diagnosis and classification for hundreds if not thousands of years already. However, there has been much variation in clinical practices even guided by the same TCM theory [4–6]. These variations are problematic when one tries to validate or replicate the effectiveness of the practice. Methods adopted from the current biomedical research to evaluate the efficacy of TCM interventions are often conceptually incompatible with the theory and clinical practice of TCM [7, 8]. Therefore, there is much need for standardized, validated instruments which can facilitate Chinese medicine diagnosis and which can be used by practitioners and researchers alike in their diagnostic process.

Tennis elbow or lateral elbow pain (LEP) is a common musculoskeletal pain condition with a prevalence of at least 1–3%. Incidence rates increase up to 10% for people between 40 and 50 years of age and symptoms are often prevailing for 1.5–2 years, therefore causing considerable loss of life quality for sufferers as well as accounting for substantial economic loss [9]. Acupuncture is frequently used to treat LEP [10].

In a previous systematic review, we have identified major TCM patterns associated with LEP. In this Delphi study, we wanted to investigate whether there is agreement between the literature and actual clinical practice. The overall aim of this study was to develop a practical instrument that will facilitate acupuncture practitioners with an easily applicable questionnaire to readily assess the underlying TCM pattern of LEP. We planned to achieve this aim through the following processes: first, we wanted to generate an initial questionnaire based on a systematic review and discussions within the research team. This preliminary questionnaire would then be presented to a Delphi panel and would undergo a Delphi survey with the following primary objectives: (1) to find consensus on which TCM patterns are the most common patterns underlying LEP; (2) to design and validate a questionnaire that would help diagnose a TCM pattern for LEP; and (3) to generate and find consensus on a list of signs and symptoms that would be indicative of one of the TCM patterns. We also used this survey to gather information for a basic list of recommended acupuncture and moxibustion treatment modalities for each pattern, as the ultimate purpose of pattern diagnosis is to guide clinical practice. This list of acupuncture/moxibustion treatment recommendations for LEP may serve as the basis for future studies.

## 2. Methods

### 2.1. Formation of Research Team

A research team, consisting of all the authors, was formed to conduct the Delphi study. The team met regularly to initially determine the aim of the pattern questionnaire and then to generate its items, to define appropriate criteria for the selection of the Delphi expert panel, to analyze and discuss quantitative and qualitative answers after both rounds, to provide appropriate feedback to the expert panel after each round, and to monitor the progress of the study.

### 2.2. Selection of Participants

Before the commencement of this study, ethical approval was obtained from the Committee on the Use of Human and Animal Subjects in Teaching and Research at the Hong Kong Baptist University, Hong Kong (reference number HASC/Student/12-13/007). Purposive sampling was used for the selection of experts. Experts were chosen with the purpose that they have knowledge and experience about acupuncture treatment for LEP, with an assumption that their knowledge about LEP signs can be used to readily determine the items in our questionnaire. We aimed to identify panelists who have a broad range of knowledge in the treatment of LEP with acupuncture and ideally previous experience of having undertaken or currently undertaking clinical research on acupuncture, including RCTs. To qualify as panelists, possible candidates were screened before entering the study for a minimum acupuncture experience of five years, had to be frequently treating LEP with acupuncture, and had to be regularly using pattern diagnosis in their clinical practice. Candidates were also asked if they had previous experience with acupuncture clinical research. Recommendations from candidates meeting these criteria for inviting additional potential panelists were taken into consideration.

National and international experts were recruited from disciplines involved in the diagnosis and treatment of LEP with acupuncture including acupuncture practitioners, acupuncture researcher, and acupuncture educators.

Prospective panelists were sent an information package via email or mail to inform them of the study goals as well as the format of a Delphi study prior to sending out the questionnaires. Immediately after the prospective panelist had agreed to participate in the study, the initial questionnaire was sent to the panelist.

Names of participating panelists are mentioned in the Acknowledgments unless they indicated otherwise. The identity of the expert panelists was disclosed to the participants before the publication of this study.

### 2.3. Generation of Items

In order to generate an initial questionnaire which was presented to the expert panelists in round 1 of the Delphi survey, information was compiled from the following sources by the research team: (a) a systematic review on TCM pattern diagnosis for LEP [11], which consisted of (i) a journal review, (ii) a textbook literature review, and (iii) a data-mining process, as well as (b) meetings of the research team, which also consisted of specialist acupuncture clinicians to acquire expert opinion and to identify relevant criteria as well as to further discuss particular questionnaire items.

Findings from these sources were collected and reviewed. The research team removed nonrelevant items and composed a preliminary questionnaire, which was divided into four sections: the 1st section stated initial possible TCM patterns that could underlie LEP. In the 2nd section, signs and symptoms, which could be clinically relevant to determine the pattern underlying LEP, were reformulated into colloquial Yes/No questions that a practitioner could address to a patient. This list of questions was divided into (i) symptoms at the local elbow area, (ii) other symptoms, and (iii) physical signs. Other symptoms were subdivided into body/limbs, digestive/stools, mind, upper body, physical signs, tongue features, and pulse features. In the 3rd section, the expert needed to decide which sign or symptom would be indicative of which pattern. And, finally, in the 4th and final section of the questionnaire the expert was asked which acupuncture/moxibustion treatment modality he or she would recommend for each pattern.

In Sections 1 and 2, the experts could choose to either agree or disagree on an item to be included in the final questionnaire. Items in Sections 3 and 4 were rated in a multiple-choice format, with multiple responses allowed (which signs and symptoms indicate which pattern and which treatment modality would be recommended for which pattern). For all four sections of the questionnaire, the experts had the chance also to choose “Other” or “Alternative method” and then could clarify their choice, in case an expert wanted to make a choice that was not provided as a default choice. At the end of each of the four sections, we included the opportunity for the experts to comment on their responses as well as to leave additional comments. The English translations of TCM patterns, pulse, and tongue features were based on the WHO standard terminologies on TCM [11].

### 2.4. Delphi Process

The Delphi method is a structured process in which consensus of opinions from a group of experts is obtained using a series of questionnaires in quasi-anonymity and with controlled opinion feedback [12]. McKenna [13] suggested that consensus in a Delphi study should be equated with 51% agreement among experts; Sumsion [14] recommended 70%, yet Green et al. [15] proposed 80% while Crisp et al. [16] challenged the idea that consensus should be equated to a percentage number and concluded that stability of the response throughout the rounds is a better index for consensus. Thus, there are no standard guidelines on an appropriate level of consensus and no apparent scientific rationale on how to decide on the degree of agreement that would amount to a consensus [17]. Based on our research on previously conducted Delphi studies, especially in the field of TCM [18–22], and due to the fact that our initial questionnaire was generated on the basis of a thorough, systematic review, the research team decided to set a content validity index (CVI) of ≥0.51. This CVI represents a minimal level of 51% of consensus among the experts and therefore a de facto majority to determinate whether or not an agreement was found between the experts and whether or not an item should remain in the next round. This consensus level was set before the start of round 1. The CVI was calculated by the number of experts who declared an item suitable divided by the total number of experts, who rated the item. We preset the amount of Delphi rounds to two, due to reasons that are further elaborated in the Discussion.

### 2.5. Round 1

In this first round, the panelists were to decide whether or not to retain an item for the final questionnaire as well as to suggest new items (i.e., a pattern, a sign or symptom, or a treatment modality that was not a default option), as previously described.

### 2.6. Round 2

Based on the results of round 1, all items with a CVI ≥ 0.51, as well as all potential new items, were presented to the experts. If an expert suggested including an additional item, it was evaluated for relevance by the research team and if it was deemed relevant, it was included in round 2. The expert panel was informed of the following: “For any item of the questionnaire to appear in round 2 at least 51% (‘the majority') of all experts had to decide to include it.” In the light of this information, the expert panel rerated all items of the four sections and was asked to either agree or disagree to retain an item for the final instrument. Experts were also informed that the second round was the final round of the Delphi survey. The final instrument would then only contain items, which consensus was reached upon after two rounds of the Delphi process.

### 2.7. Data Analysis

Data was collected and analyzed by Marcus Gadau and independently reviewed by Shi-Ping Zhang and Wing-Fai Yeung. Statistical analysis was performed using the Statistical Package for the Social Sciences (SPSS) version 21.

## 3. Results

### 3.1. Description of Expert Panel

**Figure 1:**
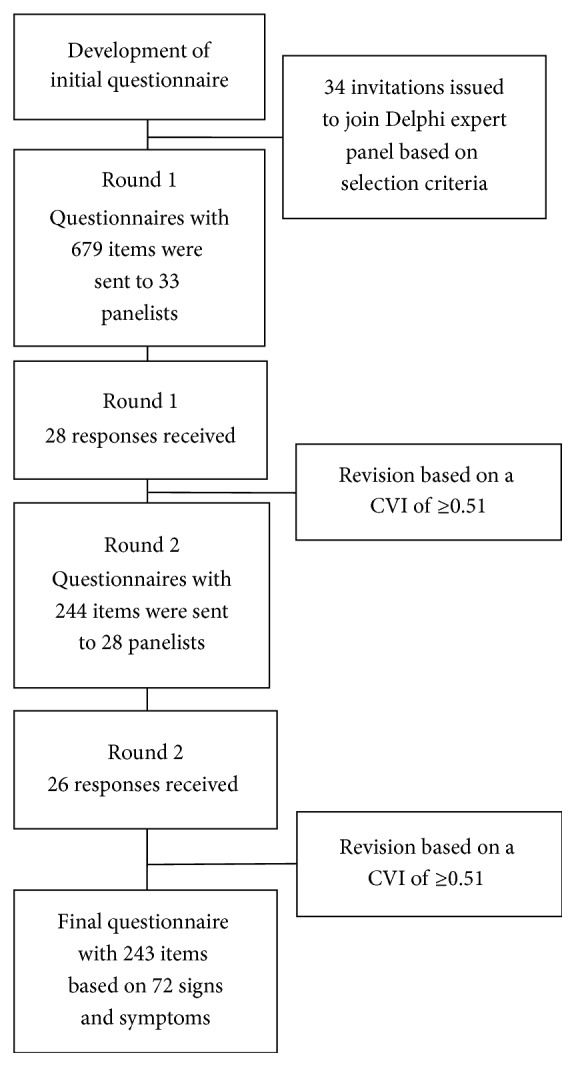
Flowchart of Delphi process.

Figure 1 shows the Delphi process. Of the 34 experts initially invited to participate in the study, 33 (97%) agreed to participate and were sent the provisional questionnaire for evaluation. One expert did not want to participate in the study, because he stopped using pattern diagnosis in his practice recently. Of the 33 experts who agreed to participate, 28 completed round 1. All 28 experts had a minimum experience of five years in the treatment of lateral elbow pain with acupuncture and had frequently been using pattern differentiation in their acupuncture practice. Finally, 26 experts completed the second round, after which the Delphi process was terminated. The reason why seven of the 33 initial panelists did not complete round 1 or round 2 is unknown to the authors, as no explanation had been given. The characteristics of the 26 panelists who completed both rounds of the Delphi study and therefore represented the expert panel are presented in Table 1.

### 3.2. Delphi Round 1

Of the original 679 items provided in the provisional questionnaire, 244 items (35.9%) remained after round 1 (Figure 1).

One additional item was added after round 1 in Section 2 of the questionnaire (see Table 3):* (Item 22) “How severely does your elbow pain affect your ability to carry out routine tasks (e.g. driving, opening jars, carrying shopping bags)? - Not at all; Mildly; Medium; Severely (answering options).”* This question was proposed by one panelist and was accepted by the research team because a question assessing the severity of functional impairment in regard to performing daily tasks was not yet presented in the original set of items and was deemed relevant for the purpose of the instrument. One expert suggested adding blood stasis as an individual pattern to Section 1 of the questionnaire (see Table 2). However, the research team did not include this item for round 2, due to findings from the previous systematic review suggesting that blood stasis appeared rather frequently in association with qi stagnation in LEP and this pattern was already included. A second expert suggested subdividing or adding in different kinds of qi- and blood-deficiencies based on the organ system they were caused by, such as spleen/stomach weakness leading to qi- and blood-deficiency. Another expert suggested subdividing or adding different channel-specific qi-stasis and blood stasis pattern such as qi-stasis and blood stasis of the hand greater Yang meridian. The research team decided not to include these patterns because they are mere subpattern of a pattern that is already included.

One expert suggested adding fire needling and another expert suggested adding distal needling acupuncture (DNA) as a recommended treatment modality to Section 4 of the questionnaire (see Table 5). Fire needling was considered too invasive and is not very commonly practiced outside of China because it is a potentially dangerous treatment modality and was therefore not included. DNA is a style of manual needling, rather than a new treatment modality, and was also not included, because manual needling includes all styles and forms of manual stimulation acupuncture, including DNA.

Other experts suggested adding tuina-massage, herbal therapy (internal and external), electromagnetic stimulus, or osteopathic therapy as recommended treatment modalities. We did not include these as the spectrum of relevant therapies for the questionnaire was preset to acupuncture practice, which must involve the use of acupoint stimulation, such as acupuncture, moxibustion, acupressure, acupotomy (scalpel therapy), auricular acupuncture, or acupressure.

### 3.3. Delphi Round 2

Expert consensus was found on 243 of 244 items from round 1 (all four sections combined). A consensus was achieved from all 26 panelists who responded to the second round on all four TCM patterns in Section 1 that could underlie LEP, namely, the* wind-cold-dampness pattern*, the* qi stagnation and blood stasis pattern*,* the dual deficiency of qi and blood pattern,* and the* retained dampness-heat pattern* (see Table 2).

The following question from Section 2 was excluded, because it did not reach a CVI of ≥0.51 in round 2:* “Does eating cold foods (i.e. watermelon, salads, icy-cold drinks) decrease the pain?”*

The final instrument that derived from a systematic review, textbook-research, and finally a two-round Delphi process was generated. It has a list of four common patterns underlying LEP (Table 2), a main-questionnaire with 72 Yes/No questions (Table 3) that can help to differentiate one of the four most common TCM patterns underlying LEP and a list of common symptoms (Table 4) that are indicative of one of the four most common patterns. A list of preferred treatment modalities (Table 5) for each of the four patterns was established as well. All items of the instrument had a CVI of at least 0.51 and therefore show considerable content validity.

## 4. Discussion

In our final TCM pattern diagnosis for LEP instrument, we identified 25 local signs and symptoms and 45 systemic signs and symptoms, as well as 16 tongue and pulse features that may be associated with the four most commonly seen LEP patterns. Even though we laid emphasis on the four most commonly seen LEP patterns during the development of this questionnaire, it can also be used in the diagnostic process of identifying a mixed pattern presentation (e.g., dual deficiency of qi and blood coexisting with wind-cold-dampness).

The experts agreed on 99.6% of all items that were presented to them in the final Delphi round, and the mean CVI of all items in the final questionnaire was 0.88 (95% CI, 0.87 to 0.90), representing an 88% consensus level. We are therefore confident that our findings adequately represent a robust consensus of TCM expert opinions. Such high agreement might be because our initial questionnaire derived from a systematic review.

We chose clinical experts rather than academic experts for our Delphi panel and the experts came from many different countries across four continents. We, therefore, may assume that our Delphi findings represent the current international notion of TCM clinical practice in regard to LEP pattern diagnosis. The high level of agreement between the literature review (academic consensus) and Delphi experts (clinical consensus) then suggests that our findings have a high degree of generalizability of LEP pattern diagnosis in TCM theory and practice. The provisional instrument created via this Delphi study has achieved considerable content validity, yet requires further face-, criterion-, and construct-validity as well as test-retest and reliability testing before it may be clinically used. We are therefore currently conducting such studies for both the English as well as the Chinese language versions of the instrument.

Even though the primary aim of the study was to provide an instrument to assist with the pattern diagnosis of LEP, we also wanted to gather information for pattern-based treatments for future studies. Therefore, we asked the experts in the last section of the questionnaire which acupuncture/moxibustion treatment modalities they would recommend for which pattern (Table 5). The average consensus level of treatment recommendations that passed the cut-off criteria of 51% expert agreement and therefore remained in round 2 was a striking 91%. Surprising to the authors, the experts did not recommend using ginger-moxibustion for the qi stagnation and blood stasis pattern; however, they did see indirect moxibustion (the use of a moxa stick held about 3 cm away from the skin) or the combined use of acupuncture and moxibustion fit for use for this pattern. Another unexpected recommendation was the use of manual acupuncture but not electroacupuncture for the dual deficiency of qi and blood pattern. An explanation from TCM theory for these two unanticipated recommendations is perhaps that both methods involve a rather strong stimulus to the injured elbow, which would be deemed contraindicated for the deficiency pattern and too “hot” or proinflammatory for the stasis pattern, which is associated with signs of eminent inflammation.

In the literature [12–27] we found that most Delphi studies used between two and four rounds to achieve consensus. Before we started to collect the data, we chose to terminate the Delphi process after two instead of four rounds out of practical reasons and due to the fact that previous research teams [25] found that the chances for low response rates increase exponentially after two Delphi rounds. A phenomenon called “response fatigue” sets in, as clinical experts are usually extremely busy with little time to spare in their tight schedules. We informed all participating experts that the Delphi study would be terminated after two rounds; this was also done to motivate participants and reduce response attrition after round 1. We also sent out regular email reminders before the deadline of each round, as well as extra reminders to experts, who have not responded after the deadline passed, also to enhance the response rate. We felt that two Delphi rounds would suffice to achieve a reliable level of consensus among experts because our initial set of items for the questionnaire derived from a systematic review and therefore would already possess some inherent level of consensus as it represents the concentrated opinions of authoritative texts.

There is a potential risk for bias in the expert selection, as the method is based on nonrandomized sampling. Therefore, representativeness cannot be assured [26]. However, we set distinct inclusion and exclusion criteria to ensure some degree of homogeneity among the experts. There are again no rules for the minimum or maximum amount of experts in a Delphi study and the choice of the size of the expert panel for most reviewed previous Delphi studies depended largely on common sense, resources and time available, and other practical reasons. Ten to fifteen subjects were deemed sufficient by Delbecq et al. [27]. We set out to have more than double the amount of experts on our panel, in case there would be a larger attrition quote. A potential risk for response bias was minimized due to the time spent educating the panel and by choosing only two Delphi rounds. Our relatively low attrition rate of 21% (7 out of 33) was probably due to these precautions taken. Experts were informed that the items presented to them in round one were generated from the literature. However, experts had ample opportunity to provide other patterns, signs, and symptoms and could comment on the items presented to them. This was done to avoid early closure on ideas and to prevent that experts alter their views due to perceived pressure to conform to the literature.

After having performed a systematic review as the basis for the initial questionnaire, we felt comfortable to choose an agreement of 51% among experts as having achieved consensus. However, we would also like to point out that the mean consensus level for all items that remained at the end of round 2 was 88% and that 85.5% of all items (208 out of 243 items) reached a consensus level of over 70%, thus making our expert consensus much more robust than a consensus just defined by a de facto majority of 51%. We attempted to address the issue of subjectivity with retained items, as another potential criticism of our study, by having chosen an expert panel with a broad range of backgrounds and geographical regions. We also believe that the very robust level of consensus that was achieved with the majority of final items has minimized the risk of such subjectivity. However, one should bear in mind that expert consensus does not automatically mean that the right answers were found. Another limitation might be that due to the selection of experts, there might be acupuncture styles practiced that were not adequately represented in our expert panel. Lastly, while interpreting the results of our Delphi study one should acknowledge the potential influence of biases and that the current preliminary instrument will have to undergo rigorous validity and reliability testing before its clinical use can be recommended.

## 5. Conclusion

While the TCM pattern diagnosis system has the potential to refine treatment by identifying subtle differences in etiology, pathogenesis, and body constitution, a lack of standardization in terminology and consensus on diagnostic criteria are significant barriers. The provisional instrument derived from our study has obtained robust consensus and could be seen as a way of controlling information variance. It has been realized that similar sets of information must be collected and standardized terminology and diagnostic criteria must be used before consensus among practitioners in regard to the TCM pattern diagnosis can be reached [28, 29]. Only with the use of standardized instruments like ours may information variance be reduced, which in combination with using a standardized terminology and diagnostic criteria could improve diagnostic-reliability as well as interrater reliability and intertrial reproducibility. This would help to overcome some of the shortcomings in TCM research and practice and represents a significant advancement in clinical TCM research [30]. Our instrument may, therefore, contribute to the standardization of TCM pattern diagnosis.

## Figures and Tables

**Table 1:** Profile of Delphi experts.

Characteristics (*n* = 26)	
Gender	
*Male*	15
*Female*	9
Acupuncture experience	
*5–10 years*	14
*11–20 years*	6
*+21 years*	5
Region	
*Asia-Pacific (incl. Australia)*	11
*Europe*	12
*North America*	2
Profession (multiple responses allowed)	
*Acupuncture practitioner*	23
*Acupuncture researcher*	13
*Acupuncture educator*	17

**Table 2:** Patterns associated with LEP.

Item	Chinese name	Experts that agree % (*n* = 26)
(1) Wind-cold-dampness pattern	*风寒*湿证	100
(2) Retained dampness-heat pattern	湿热内蕴证	77
(3) Dual deficiency of qi and blood pattern	气血两虚证	87
(4) Qi stagnation and blood stasis pattern	气滞血瘀证	100

**Table 3:** Final LEP pattern differentiation questionnaire.

Item	Experts agree % (*n* = 26)
Local symptoms:	
(1) Does your elbow feel cold?	88
(2) Does your elbow feel hot?	92
(3) Does cold exposure increase the pain?	100
(4) Does cold exposure relieve the pain?	80
(5) Does heat exposure increase the pain?	80
(6) Does heat exposure relieve the pain?	96
(7) Do you strongly dislike cold on your elbow?	92
(8) Do you strongly dislike wind on your elbow?	78
(9) Do you strongly dislike heat on your elbow?	71
(10) Does local pressure increase the pain?	100
(11) Does local pressure relieve the pain?	88
(12) Does movement increase the pain?	100
(13) Does movement relieve the pain?	88
(14) Does rest increase the pain?	84
(15) Does rest relieve the pain?	100
(16) Which term best describes your pain?	100
*Dull, lingering *	92
*Cramping *	100
*Stabbing *	96
*Hot, burning *	88
*Numbness sensation*	88
(17) How severe is your pain?	100
*Mild*	96
*Medium *	100
*Severe *	100
(18) Is there any uncomfortableness during movement?	96
*None *	95
*Mild*	95
*Medium *	96
*Severe *	95
(19) How was the onset of your elbow pain?	100
*Slow, gradual onset*	100
*Sudden onset*	100
(20) How long have you had the condition?	100
*More than 3 months*	100
*1 week–3 months*	100
*Less than 1 week*	100
(21) Is the pain intermittent or constant?	100
*Intermittent *	100
*Constant *	100
(22) How severely does your elbow pain affect your ability to carry out routine tasks (e.g. driving, opening jars, carrying shopping bags)?	100
*Not at all*	95
*Mildly*	96
*Medium*	100
*Severely*	100

Systemic symptoms:	
(23) Do your hands and feet usually feel cold?	92
(24) Do your entire arms and legs usually feel cold?	88
(25) Do your hands and feet usually feel too hot?	67
(26) Are your hands and feet usually sweaty?	74
(27) Do you often experience spontaneous sweating over your entire body?	78
(28) Does your entire body usually feel heavy?	80
(29) Do you usually have a feeling of fullness, especially in the epigastric region (upper area of the belly)?	67
(30) Do you usually feel full or bloated after eating?	75
(31) Do your arms and legs usually feel weak?	76
(32) Do you experience numbness in your arms and legs?	80
(33) Does eating cold foods (e.g. watermelon, salads, ice-cold drinks) increase the pain?	58
(34) Does eating warm food (e.g. chili, pepper, ginger, hot soups) increase the pain?	54
(35) Does eating warm food (e.g. chili, pepper, ginger, hot soups) decrease the pain?	68
(36) Do you usually have poor appetite?	61
(37) Do you usually have excessive appetite?	57
(38) Do you usually feel thirsty?	63
(39) Do you usually feel thirsty, but you do not want to drink?	67
(40) Are you usually not thirsty at all?	67
(41) Do you usually have loose stools?	67
(42) Do you usually have dry stools?	63
(43) Do you usually have copious, clear urine?	71
(44) Do you usually have scanty, dark-yellow urine?	63
(45) Do you have urinary difficulties?	52
(46) Do you usually feel tired and easily fatigued?	79
(47) Do you usually feel restless and/or agitated?	74
(48) Do you have difficulties sleeping and/or shallow sleep	78
(49) Do you frequently experience mood changes?	70
(50) Do you easily get angry?	83
(51) Are you usually worried and/or anxious?	70
(52) Does your head usually feel heavy?	67
(53) Do you usually feel dizzy?	61
(54) Do you usually have a lot of saliva in your mouth?	55
(55) Do you usually experience a bitter taste in your mouth, especially in the morning?	61
(56) Do you usually have a sticky taste in your mouth?	65
(57) Are you usually short of breath?	70

Physical signs:	
(58) The elbow feels cold to touch (to the practitioner)?	96
(59) The elbow feels warm to touch (to the practitioner)?	92
(60) The elbow is swollen and/or reddened?	92
(61) Does the face appear pale and/or lusterless?	87
(62) Does the complexion appear oily?	73
(63) Does the complexion appear reddened and/or dry?	74
(64) Does the complexion appear dark?	74
(65) Do the lips appear brittle?	70
(66) Do the lips appear purple?	83
(67) Do the lips appear excessively red?	65
(68) Do the nails appear brittle?	74
(69) Does the voice appear strong?	65
(70) Does the voice appear soft?	74

Tongue and pulse features:	
(71) Tongue features	91
*Pale tongue*	86
*Red tip of the tongue*	76
*Red tongue*	78
*Scanty fur*	77
*Slimy fur*	78
*Thick fur*	76
*Thin fur*	86
*White fur*	87
*Yellow fur*	77
(72) Pulse features	92
*Fine pulse*	91
*Rapid pulse*	82
*Slippery pulse*	77
*String-like pulse*	91
*Sunken pulse*	86
*Replete (strong) pulse*	82
*Weak pulse*	87

**Table 4:** Patterns and their indicating signs and symptoms.

Item	Experts agree % (*n* = 26)
*(1) Wind-cold-dampness pattern*	
(1) Elbow feels cold to the patient	100
(2) Cold exposure increases the pain	100
(3) Heat exposure relieves the pain	96
(4) Patient strongly dislikes cold on the elbow	96
(5) Patient strongly dislikes wind on the elbow	83
(6) Local pressure increases the pain	63
(7) Movement increases the pain	79
(8) Movement relieves the pain	75
(9) Rest relieves the pain	68
(10) Nature of pain: Dull/lingering	87
(11) Nature of pain: Cramping	88
(12) Nature of pain: Numbness sensation	96
(13) Pain severity: Medium	88
(14) Pain severity: Severe	92
(15) Uncomfortableness during movement: Medium	79
(16) Onset of elbow pain: Slow, gradual onset	72
(17) Onset of elbow pain: Sudden onset	80
(18) Duration of condition: 1 week–3 months	88
(19) Duration of condition: Less than 1 week	76
(20) Intermittent or constant pain: Constant	92
(21) Hands and feet usually feel cold (to the patient)	79
(22) Entire body usually feels heavy	68
(23) Eating warm food (e.g. chili, pepper, ginger, hot soups) decreases the pain	71
(24) Loose stools	68
(25) Copious, clear urine	79
(26) Patient usually feels tired and is easily fatigued	82
(27) Elbow feels cold to touch (to the practitioner)	96
(28) Pale tongue	91
(29) White tongue fur	96
(30) Slippery pulse	86
(31) String-like pulse	83

*(2) Retained dampness-heat pattern*	
(1) Elbow feels hot to the patient	100
(2) Cold exposure relieves the pain	86
(3) Heat exposure increases the pain	91
(4) Heat exposure relieves the pain	52
(5) Patient strongly dislikes heat on the elbow	82
(6) Local pressure increases the pain	91
(7) Movement increases the pain	86
(8) Nature of pain: Hot/burning	100
(9) Pain severity: Medium	82
(10) Pain severity: Severe	95
(11) Uncomfortableness during movement: Medium	86
(12) Uncomfortableness during movement: Severe	90
(13) Duration of condition: 1 week–3 months	95
(14) Intermittent or constant pain: Constant	91
(15) Scanty, dark-yellow urine	82
(16) Patient usually feels restless and/or agitated	65
(17) Sensation of heaviness of the head	57
(18) Usually bitter taste in the mouth, especially in the morning	71
(19) Usually a sticky taste in the mouth	76
(20) Elbow is swollen and/or reddened	95
(21) Red tongue	91
(22) Thick tongue fur	77
(23) Yellow tongue fur	86
(24) Rapid pulse	91
(25) Slippery pulse	91
(26) Replete (strong) pulse	67

*(3) Dual deficiency of qi and blood pattern*	
(1) Elbow feels cold to the patient	90
(2) Cold exposure increases the pain	95
(3) Local pressure relieves the pain	90
(4) Movement increases the pain	86
(5) Rest relieves the pain	90
(6) Nature of pain: Dull/lingering	95
(7) Nature of pain: Numbness sensation	90
(8) Pain severity: Mild	95
(9) Pain severity: Medium	81
(10) Onset of elbow pain: Slow, gradual onset	95
(11) Duration of condition: More than 3 months	95
(12) Intermittent or constant pain: Constant	86
(13) Hands and feet usually feel cold (to the patient)	81
(14) Spontaneous sweating	70
(15) Limbs usually feel weak	81
(16) Numbness sensation in limbs	81
(17) Poor appetite	70
(18) Loose stools	75
(19) Patient usually feels tired and is easily fatigued	86
(20) Patient usually feels dizzy	60
(21) Elbow feels cold to touch (to the practitioner)	81
(22) Pale, lusterless face	90
(23) Soft voice	80
(24) Pale tongue	95
(25) Thin tongue fur	86
(26) White tongue fur	90
(27) Fine pulse	95
(28) Sunken pulse	75
(29) Weak pulse	95

*(4) Qi stagnation and blood stasis pattern*	
(1) Local pressure increases the pain	96
(2) Movement relieves the pain	83
(3) Nature of pain: Stabbing	100
(4) Nature of pain: Numbness sensation	77
(5) Pain severity: Medium	87
(6) Pain severity: Severe	100
(7) Uncomfortableness during movement: Mild	73
(8) Uncomfortableness during movement: Medium	96
(9) Onset of elbow pain: Slow, gradual onset	79
(10) Duration of condition: More than 3 months	79
(11) Duration of condition: 1 week–3 months	79
(12) Intermittent or constant pain: Intermittent	83
(13) Intermittent or constant pain: Constant	82
(14) String-like pulse	92

**Table 5:** Treatment recommendations.

Treatment intervention	Patterns and agreement of experts % (*n* = 26)			
	Wind-cold-dampness pattern	Retained dampness-heat pattern	Dual deficiency of qi and blood pattern	Qi stagnation and blood stasis pattern
Acupuncture	92	91	96	96
*Manual acupuncture*	92	91	91	83
*Electro-acupuncture*	88	—	—	96
Moxibustion	92	—	91	87
*Ginger-moxibustion* (direct moxibustion with a thin slice of ginger between skin and moxa cone)	88	—	81	—
*Moxa stick* (indirect moxibustion about 3 cm away from elbow)	96	—	91	88
Acupuncture and moxibustion	96	—	95	92

—, treatment modality CVI < 0.51: experts do not recommend this treatment modality.
